# Remarks of Prof. J. McF. Gaston upon Opening the Course in Surgery at the Southern Medical College*Stenographically reported by Mr. C. A. Smith, a member of the graduating class.

**Published:** 1897-11

**Authors:** 


### Southern Medical Record.

A MONTHLY JOURNAL OF MEDICINE AND SURGERY.

VOL. XXVII. ATLANTA, GA., NOV., 1897. No. 11

### Original Articles.

### REMARKS OF PROF. J. McF. GASTON, UPON OPENING THE COURSE IN SURGERY, AT THE SOUTHERN MEDICAL COLLEGE.*

We propose to take up this morning a general consideration of surgery, and it is my privilege and duty to teach the principles and practice of surgery in contradistinction to operative surgery. The principles of surgery are very important as a guide to you always in reference to your adoption of rules for carrying out your operations in surgery. The principles of surgery cover a very large field, but many think that there is very little attached to surgery and that the cutting is all that there is to be learned. I want to discourage any ideas of this kind; we do not want to make mere "cutters." We want you to have some ideas and know what you want to do. I consider diagnosis and prognosis often more than the mere cutting. At the same time we would have you skillful operators, with as much dispatch and skill as it is possible to possess. In the present day by the use of anæsthesia it might be supposed to be of little consequence as to the amount of time consumed in an operation. However, it is of much importance to use dispatch where it is possible; we should not delay our operation because we have the patient under the anæsthetic. I have no doubt that patients have in many instances succumbed under the effects of the prolonged anæsthesia. Therefore, gentlemen, we would impress upon you the importance of understanding the principles which underlie the practice of surgery, and in the course of lectures

*Stenographically reported by Mr. C. A. Smith, a member of the graduating class.

which we have to present to you we will make it a point to present those views which are clearly recognized by the profession and which are generally used, but I do not propose to throw aside old views which are entirely correct and proper. I shall give you these views where I think they are beneficial, but at the same time shall expect you to understand the why and wherefore of such procedures.

It is important to understand what is meant by surgery, and my mode of presenting the subject is to draw a clear distinction between what is understood as internal and external medicine. We especially would insist that we regard surgery as pertaining to the structural and organic changes, in contradistinction to functional derangements which are in the practice of medicine. This is the groundwork of distinction between the two, and I would claim that any change which is perceptible to the eye and touch is a surgical disease and should be treated under its principles, and not simply by mechanical means, but even medicinal measures may be brought into use, both before and after an operation, to sustain the powers of the patient. So that all that is necessary to be done in connection with a case of organic derangement is under the treatment of surgery. We would not expect a surgeon to send out and get a physician to treat such symptoms as may arise in a surgical case. In fact, many diseases are getting to be amenable to medicinal measures which were not previously so considered or recognized. As you are aware, the standard treatment now of diphtheria (which is really a surgical condition in connection with organic changes) is now found to be amenable to the anti-toxine treatment. I think the lights on the subject should satisfy us that the anti-toxine method is radical in the treatment of diphtheria. And so it is with a great many other complications in this line, and just now we have one coming to the front which does not belong to surgery, and this is in reference to the treatment of yellow fever. Sanarelli, the Italian, claims to have discovered a bacillus which will serve as an antidote to yellow fever. Some of you are aware of the interest we have taken in this line. Domingos Freire, of Rio, claimed to have discovered the bacillus of yellow fever and he inoculated people and has had favorable results. So we find that treatment in the progress of science is undergoing many changes. Large fields of knowledge are now open to you which were not open to me when I studied medicine. Any student who graduates from our class this year will be better qualified in surgery than the professors were of fifty years ago. I remember when I went in to hear the old lecturers in the University of Pennsylvania we were accustomed to look upon them as oracles in everything, but if one of them were to appear before you to-day and endeavor to instruct you they would be wanting in a great deal.

The great progress that has been made in surgery in connection with the different regions of the body is a very important thing to point out to you at this stage. In former times very little was known about brain and scalp surgery as compared with what is known at the present day. You are aware that by the investigations of Horsley and others that the brain centers corresponding to the distribution of the different nerves have been so well defined that diagnosis of brain involvment can be quickly made from the peripheral nerves involved. We can not always say what is the cause of the trouble but can decide upon the region of the brain involved by the affection of the remote distribution of the nerves; not only the extravasation of blood but other forms of trouble involving local disturbances of the brain which are involved in the injury or are the result of disease. However, there is always and always will be, the question as to where a trephine should be applied in ordinary injuries of the brain, because the blow expends itself at a point distant from the immediate proximity of the injury; as for instance, a blow upon the vertex producing fracture of the base of the skull. But at the present time, even those injuries which involve the base of the brain have been sought to be relieved by surgical appliances.

In a like manner, in the chest, great advances have been made in connection with disorders of the thoracic region. We are all familiar with those cases in which serous accumulations occurred in the cavity of the chest, but there was a considerable degree of negligence or of ignorance in connection with the transformation of serum into purulent conditions of the chest, and the advancement of late years has been especially with reference to empyæma. The various methods used to relieve this and the approximation of the pleural lining of the chest wall will be treated further on.

Likewise, in the abdominal region we have had revelations within the last quarter of a century. From the first opening of the abdominal cavity by McDowell it has now become a very common thing. Taite, of England, says that if he has any abdominal trouble which he can not diagnose thoroughly that he will go into the abdomen to find out. But a great many of our abdominal surgeons are so acute as to diagnosis that they rarely ever make a mistake. If one of you were called into a case of abdominal trouble you perhaps would not be able to diagnose it, but not so with the abdominal surgeon. These cases are usually amenable to cutting operations, but in some instances they have been treated by other means. Even cases of neoplasm have been found amenable to local treatment. I have now in my practice a case of sarcoma of the abdominal cavity in which two surgeons had made exploratory operations; that is, one cut into the cavity expecting to find something else and when he found the sarcoma he immediately dropped the case, and the other one expected to find this and took sections for microscopical examination and fixed upon the diagnosis as small-celled sarcoma and declined to operate further and gave an unfavorable prognosis. I was called in to see the case and when I saw nothing could be done by operating, I decided to use electrolysis. This consists of passing an electric current through the growth so as to produce changes in the cells. Cataphoresis was employed by applying medicine to one pole and carrying it into the system by the current. This line of treatment is a new one. Dr. B. Massey has adopted a somewhat similar line, and I entered upon my case without knowing anything about his method. We know that Apostoli's treatment has been of some good. Yet the operators in abdominal surgery who have not satisfactory results by this method say that it leaves adhesions which render matters worse for operating. However, this method applies only to those cases which are non-operable, and all such cases may be treated with electricity with the hope of having satisfactory results.

We have troubles involving the nervous system which are now treated by surgical means and which were formerly left to medical treatment without any radical effect. Some have resorted to nerve stretching for neuralgia and in some instances have had good results. In other cases of neuralgia the division of the nerve and taking out a portion has been resorted to, and more especially the gasserian ganglion has been removed by surgeons for facial neuralgia.

We find that operative measures which have been resorted to in connection with the vascular system have been accompanied by good effect. By this we do not mean the ligation of arteries, etc., for a laceration, but the operations which have been resorted to for the cure of aneurisms and other such troubles. Also, Dr. Murphy, the inventor of the Murphy button, has experimented upon animals and has united the arteries after they have been cut, and he has also been successful in one or two cases upon human subjects and restored walls of the arteries; and also where an artery has been torn it has been sectioned and reunited. He did this in one case of the brachial artery.

These advances are far beyond what we would have expected years ago. It has been in these latter days the habit of surgeons and physicians to look for an explanation of the troubles connected with the physical condition to the introduction of germs in some form, and this theory has instituted a great revolution in the matter of treatment. I think it has been instrumental in doing more for surgery than general medicine. However, in some classes of troubles it has been proven that it has no connection. For instance, Dr. Senn, who is one of the promoters of the germ theory, claims in his definition of tumors that germs have no connection with them, and he also states that in some cases where they are associated with the disease that they are simply the accompaniment of the disease without being the cause, and in some instances we have proof that the disease is not connected with the germs as an original factor. In some instances they are the result of ptomains. Koch has proven that the tubercle bacillus can be introduced and in certain conditions the disease would be produced. Based upon this idea a curative method was advanced by Koch some years ago and it was at first supposed that a safe method had been discovered. But it was found that the tuberculin of Koch was capable of doing harm and in a very few cases it was found to be of some value. Koch is now investigating another tuberculin. The old tuberculin is now being used to discover the disease in cattle. If we can only arrive at the point that we can certainly get the germ which developes the form of disease and the secret of phagocytory action, or the anti-toxine action by which the agency can be controlled, we will have accomplished the most important result that can be done in surgery or medicine.

Naturally there comes in connection with the consideration of the germ origin of the disease the question of aseptic surgery. In connection with aseptic and antiseptic surgery I have been accustomed to include septic measures. Instead of using germicides, etc., I have advocated the cleanly or the present septic method. Those views which I advanced alone for fifteen years have at last been brought forward by the bacteriologists of to-day. There is a place for anti-septic surgery. The application of germicides in normal structures was improper, but where you have an indication of septic process then is the time for the use of the germicides, or antiseptics. This, however, was not the idea of anti-septic surgery as inculcated by Lister. His idea was that there were germs which floated in the air and which produced the harm in the wound and therefore the antiseptics were used in all operations. However, this is not now accepted. Therefore, corrosive sublimate is not used as an application to normal structures, but where you have a septic process then you would be warranted in using antiseptic methods. This is the basis upon which I have been fighting for the last fifteen years.

This subject of antiseptic surgery is one that requires more than passing notice, as it is worth the most mature observation. It does not depend upon the atmospheric condition around the patient but it is found that the fingers themselves are the vehicles for conveying the septic properties, and also the needles and threads, and therefore it is necessary that we should have cleanliness at all times, and especially in reference to the hands. It is a remarkable observation in this matter that after washing with the nail brush and soap, and even after using the corrosive sublimate, a septic matter is also left.

